# Light scattering corrections to linear dichroism spectroscopy for liposomes in shear flow using calcein fluorescence and modified Rayleigh-Gans-Debye-Mie scattering

**DOI:** 10.1007/s12551-018-0458-8

**Published:** 2018-09-25

**Authors:** Glen Dorrington, Nikola P. Chmel, Stephen R. Norton, Alan M. Wemyss, Katherine Lloyd, D. Praveen Amarasinghe, Alison Rodger

**Affiliations:** 1 Department of Chemistry, University of Warwick, Coventry, CV4 7AL UK; 2 MOAC Centre for Doctoral Training, University of Warwick, Coventry, CV4 7AL UK; 3 Department of Molecular Sciences, Macquarie University, Sydney, NSW 2109 Australia

**Keywords:** Scattering, linear dichroism, liposomes, Rayleigh-Gans-Debye, Mie Scattering, calcein

## Abstract

The interpretation of data from absorbance spectroscopy experiments of liposomes in flow systems is often complicated by the fact that there is currently no easy way to account for scattering artefacts. This has proved particularly problematic for linear dichroism (LD) spectroscopy, which may be used to determine binding modes of small molecules, peptides and proteins to liposomes if we can extract the absorbance signal from the combined absorbance/scattering experiment. Equations for a modified Rayleigh-Gans-Debye (RGD) approximation to the turbidity (scattering) *LD* spectrum are available in the literature though have not been implemented. This review summarises the literature and shows how it can be implemented. The implementation proceeds by first determining volume loss that occurs when a spherical liposome is subjected to flow. Calcein fluorescence can be used for this purpose since at high concentrations (> 60 mM) it has low intensity fluorescence with maxima at 525 and 563 nm whereas at low concentrations (<1 mM) the fluorescence intensity is enhanced and the band shifts to 536 nm. The scattering calculation process yields the average axis ratios of the distorted liposome ellipsoids and extent of orientation of the liposomes in flow. The scattering calculations require methods to estimate liposome integrity, volume loss, and orientation when subjected to shear stresses under flow.

## Introduction

**Fig. 1:**
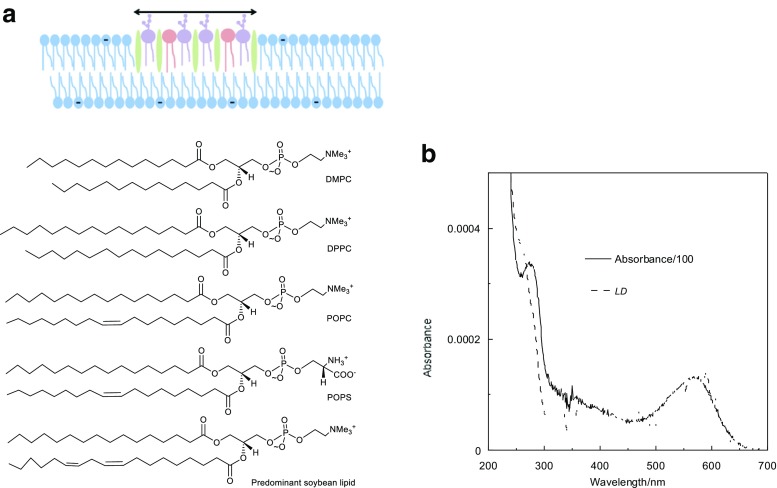
**a** Schematic of a lipid bilayer and examples of lipids (available from Avanti Polar Lipids Inc., Alabaster, AL, USA) which can form bilayers. DMPC, DPPC, POPC and POPS denote, respectively: 1,2-dimyristoyl-sn-glycero-3-phosphocholine, 1,2-dipalmitoyl-sn-glycero-3-phosphocholine, 1-palmitoyl-2-oleoyl-sn-glycero-3-phosphocholine and 1-palmitoyl-2-oleoyl-sn-glycero-3-phosphoethanolamine. **b** Absorbance and LD spectra of bacteriorhodopsin (0.2 mg/mL) with soybean (0.5 mg/mL) liposomes at pH 7. 0.5 mm pathlength, 3000 rpm. Data from reference (Rajendra et al. 2006)

Liposomes are spherical lipid bilayers (Fig. 1) that encapsulate an aqueous environment. They have been commonly used for over a decade as simple models for cell membranes (Alves et al. 2017; Beevers et al. 2010; Jesorka and Orwar 2008; Kamiya and Takeuchi 2017; Maheux et al. 2016) and they are increasingly used as environments for membrane protein studies. Some lipids used to make liposomes for biophysical experiments are illustrated in Fig. 1a. When liposomes of a size comparable to the wavelength of light are used in optical spectroscopy experiments, the absorbance signal is usually conflated with scattering because the experiments involve measuring the flux of photons that do reach the detector—regardless whether photons are lost due to absorbance or scattering. The problem is illustrated by the absorbance and linear dichroism (LD) data for bacteriorhodopsin in soybean liposomes in Fig. 1b, where it looks as if the baseline is sloping upwards as one proceeds to lower wavelength. However, exactly what the scattering contribution to the signal at 250 nm is hard to guess. Thus to use liposome spectroscopic data quantitatively, a scattering correction is required. Relatively simple correction approaches, such as using principal component analysis, have been used with some success to correct spectroscopic data for scattering artefacts with stationary particles (Vermeer et al. 2016). However, less success has been had with flow *LD* experiments that involve both polarised light and non-spherical particles. This article integrates a review of the literature covering the theoretical frameworks for scattering analysis, and outlines and implements how it can be applied in practice to extract absorbance spectra from liposome LD measurements*.* A roadmap for the approach adopted is first to use calcein leakage experiments to measure the volume loss when the spherical liposome is distorted in flow. Then, it is possible to use the volume loss and the scattering signal outside an absorbance region to estimate shape of the flow-distorted liposome from which we can calculate the orientation parameter for the LD experiment. An estimate of the surface area (or lipid density) change is also determined.

## Linear dichroism

**Fig. 2:**
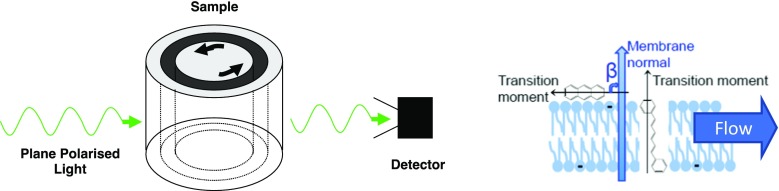
Experimental Couette flow schematic and the predominant local orientation of a lipid bilayer in the cell

LD is a differential absorption technique performed on oriented samples (Nordén 1978; Nordén et al. 2010)

1 $$ LD={A}_{\Big\Vert }-{A}_{\perp }={A}_Z-{A}_Y $$

where ∥ and *z* denote the sample orientation direction (the direction of flow) (Ardhammar et al. 2002; Ardhammar et al. 1998; Johansson et al. 1978; Johansson et al. 1976; Matsuoka and Nordén 1983; Michl and Thulstrup 1986; Nordén 1975; Nordén 1978; Nordén 1980; Nordén et al. 1992; Nordén et al. 2010; Samori and Thulstrup 1988; Thulstrup and Michl 1989; Thulstrup et al. 1970). Usually only *A*_LD_ (absorbance LD) is considered and expressed in terms of the polarisation of the transition moment and the orientation parameter, *S*,(Nordén 1978; Nordén et al. 2010)

2 $$ {A}_{\mathrm{LD}}=\frac{3}{2}S{A}_{\mathrm{iso}}\left(3{\cos}^2\zeta -1\right) $$

where *A*_iso_ is the isotropic absorbance and *ζ* is the angle between the molecular orientation axis, *z*, and the transition moment (direction of net electron displacement within the molecule). For liposomes where the analytes of interest are usually oriented normal to the bilayer (Ardhammar et al. 1998; Rodger et al. 2002), we use the alternative equation

3 $$ {A}_{\mathrm{LD}}=\frac{3}{4}S{A}_{\mathrm{iso}}\left(1-3{\cos}^2\beta \right) $$

where *β* is the angle between the membrane normal and the transition moment (Fig. 2). The challenging part of LD analysis is usually to determine *S*. If we can determine the dimensions of the flow-distorted liposomes and if they orient like rigid rods in flow, our previous work (McLachlan et al. 2013a) can be used to find *S*.

## Liposome preparation

Many methods that are variations on a theme have been established to produce liposomes of fairly uniform size (Allen and Cleland 1980b; Ardhammar et al. 1998; Maheux et al. 2016; Rajendra et al. 2006). One approach is to dissolve the lipids (and any probes that are desired to be incorportated into the lipid bilayer) in chloroform, then spread them out in a thin film on the inside of a round-bottomed flask by removing the chloroform using a rotary evaporator and a desiccator under vacuum. When the buffer of choice (e.g. sodium phosphate 10 mM, pH 7.4) is added to a final concentration of ~ 20 mg/mL lipid and the flask sonicated, the result is a wide range of sizes of spherical lipid bilayer particles (liposomes) with buffer inside. Two or three freeze-thaw cycles, using dry ice and ethanol to achieve a flash-freeze at approximately − 78 °C, followed by a room temperature thaw creates a suspension of large liposomes which can be reduced to a fairly uniform size by extrusion (typically 11 or 13 times) through a polycarbonate membrane with the desired pore size (typically 100 nm). Dynamic light scattering (using a nano-series Zetasizer, Malvern, UK) usually indicates a narrow distribtuion about 100 nm (or slightly larger) after extrusion (Damianoglou et al. 2010).

Diphenylhexatriene (DPH) (Wemyss et al. 2018) (1% *w*/*w*) works quite well as an integral probe added to the lipids at the start of the liposome preparation. It is sometime also desireable to fill liposomes with a different solution than is outside the liposome. Unless liposomes are particularly leaky (see below), this can be achieved by adding the desired molecule to the resuspension buffer and running the extruded liposome solution down a sepharose column 4B column (size 2.5 cm × 5 cm, Sigma-Aldrich) and collecting the second coloured band to be eluted off the column in a mobile phase of EDTA (0.1 mM), TES (10 mM), and NaCl (100 mM).

If the liposomes remained spherical when subjected to shear flow, they will not have an LD signal. So, the observation of an LD signal shear flow is switched on means the liposomes are distorted. Key parameter for understanding what happens to liposomes in flow is therefore their structural integrity and the volume change when flow is switched on and off. How much volume leaks out of the liposomes gives us information about both aspects. Calcein is a fluorophore that has low intensity fluorescence at high concentration (> 60 mM, see below) whereas at low concentrations (< 1 mM) the intensity is significant. This means that vesicles filled with calcein will have only very low fluorescence, but, if the calcein leaks out, the fluorescence switches on and its wavelength maximum and intensity can be used to measure how much has leaked. Vesicles can be prepared containing 50 mM calcein by adding calcein to the resuspension buffer. After extrusion to the required size, external calcein can be removed by size-exclusion chromatography to exclude free dye.

## Liposome LD

It has been apparent from the initial discovery by Ardhammar et al. (Ardhammar et al. 1998) of the fact that molecules bound to liposomes in a Couette flow cell give rise to a flow LD signal that scattering is a significant contributor to the observed signal so

4 $$ {\mathrm{LD}}_{Measured}={A}_{\mathrm{LD}}+{\tau}_{\mathrm{LD}} $$

where *A*_LD_ is the true absorbance LD and *τ*_LD_ is the so-called turbidity LD which is the signal resulting from photons that do not reach the detector because they are scattered (Nordh et al. 1986).

**Fig. 3:**
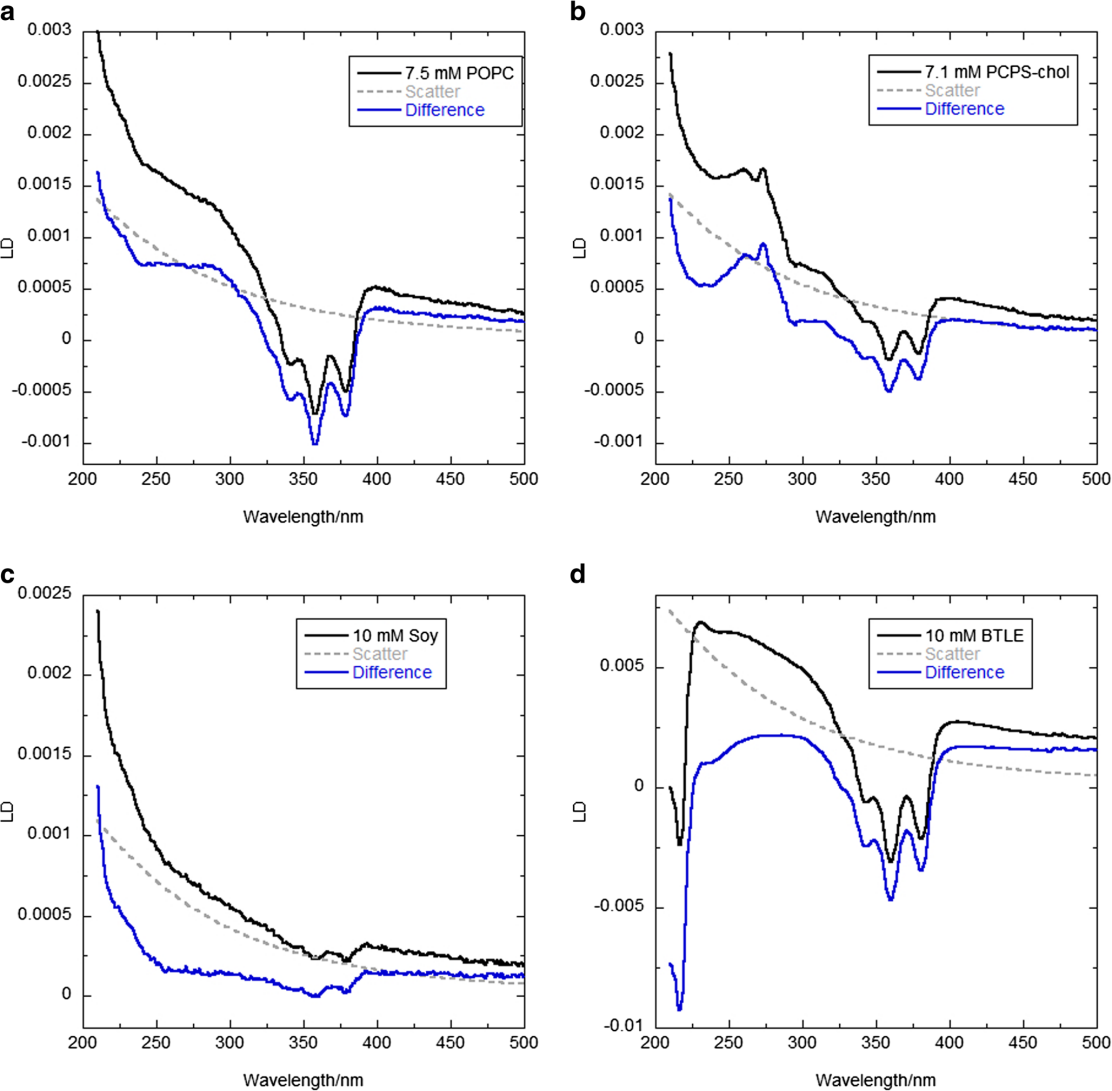
Representative LD spectra (black, upper curves) from 100 nm liposome samples containing DPH (1% *w*/*w*, see text for preparation methods) collected with a Jasco J-815 circular dichroism spectropolarimeter adapted for linear dichroism spectroscopy using a microvolume Coutette flow cell (Crystal Precision Optics, Rugby, UK) rotated at 0 rpm or 3000 rpm. **a** POPC (7.5 mM), **b** POPC/POPS/cholesterol (total concentration 7.1 mM, mixed at a 75:10:15 ratio), **c** soybean PC (10 mM) and **d** BTLE (10 mM) spectra. Soy denotes a polar extract from soybeans (mainly phosphoethanolamines (PE), phosphocholines (PC) and phosphoinositols (PI)); and BTLE refers to brain total lipid extract from Avanti Polar Lipids Inc. Scattering curves (dashed lines, see below for calculation methodology) are determined assuming the liposome surface area remains constant with onset of flow. The difference between the experimental data and the scattering curve are shown in blue (lower curves)—making the absorbance LD signal. Pathlength is 0.5 mm

The steep slopes of the low wavelength end of the measured LD spectra of Fig. 3 illustrate the significance of scattering artefacts in 100-nm liposome LD spectroscopy. From the perspective of flow-aligned spectroscopic techniques—in this work the focus is on linear dichroism (LD)—the more we understand about the response of liposomes to an induced shear flow, the more information can be extracted about how lipid-binding small molecules, peptides, and proteins operate and align on or in a membrane. It should be noted that the BTLE (brain total lipid extract) sample has significant (oriented) absorbers at 220 nm and below that are not present in the purer samples.

Nordén et al.(Ardhammar et al. 2002) reduced with the scattering problem for liposome LD by matching the refractive indices of the lipid vesicles and the surrounding medium via the addition of sucrose, thus lowering the *relative* refractive indices of the liposomes and their environment. This allowed them to measure an accurate true absorbance LD spectrum of membrane-oriented tryptophan down to ~ 220 nm (much of which was previously obscured by scattering). Unfortunately the refractive index-matching approach does not solve the scattering interference problem at lower wavelengths where sucrose absorbs. Using microvolume Couette flow cells (outer rotating quartz capillary ~ 250 μm from a stationary 3 mm quartz rod, Dioptica Scientific Ltd., Rugby, UK) (Marrington et al. 2005; Marrington et al. 2004) where the sample holder is a curved quartz capillary/quartz rod reduces scattering by acting as an additional focusing lens helps reduce the scattered light that does not reach the detector. However, it does not remove it completely.

## Liposome shapes in flow

Before reviewing scattering equations derived in the literature of relevance to this problem and illustrating how they can be applied, we need to understand the shapes that liposomes adopt in flow. The fact that liposomes orient in flow means they are not spherical. Marmottant et al. undertook an analytical study of small vesicle deformation for arbitrary flow fields (Marmottant et al. 2008) following the usual assumption that liposomes distort into some kind of ovoid shape in flow. Using phase contrast microscopy of giant (> μm diameter) vesicles Mader et al. (2006) visualised the shape of shear distorted liposomes as ellipses of about 2–3:1 axial ratio and considered their tank-treading and tumbling motion based on the theoretical work of Keller and Skalak (1982). We have used the simpler model of a cylinder with hemispherical caps to model liposomes in previous work (Rodger et al. 2002). For all distortions from spherical, liposome surface area or internal volume or both must change when a solution of liposomes is made to flow. So step 1 for LD analysis is to determine what volume leaks out of liposomes when they are subject to flow distortion.

## Calcein fluorescence calibration

**Fig. 4:**
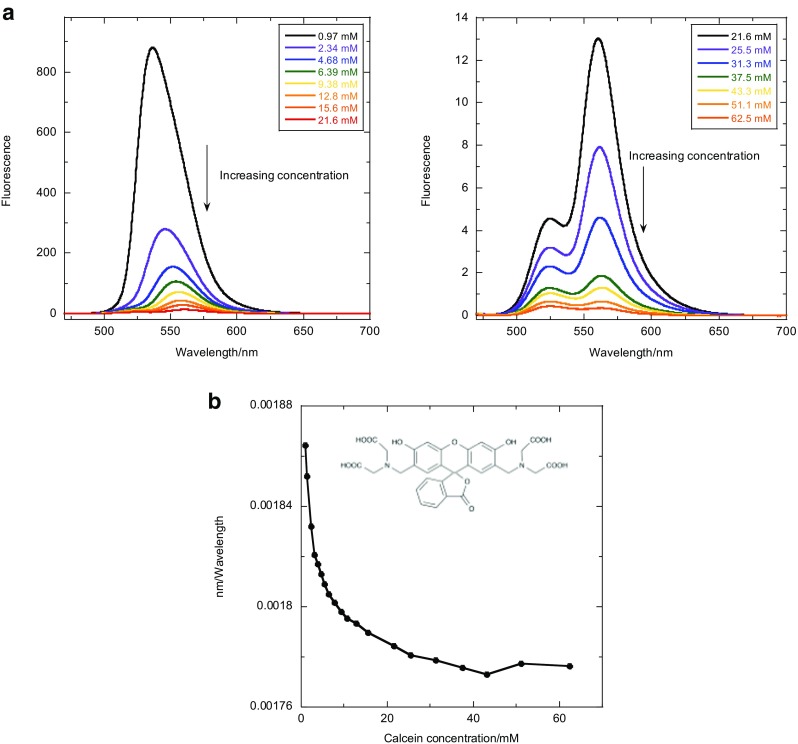
**a** Emission spectra for calcein excited at 460 nm as a function of concentration. **b** Concentration of calcein vs maximum of emitted fluorescence

Calcein fluorescence is often used to determine whether membranes leak their contents as calcein at high concentration has no fluorescence due to self-quenching yet has significant fluorescence when diluted upon leaking out of the liposome (Allen and Cleland 1980a, b). It is a simple step to use this approach to measure how much solution volume leaks out of liposomes in LD experiments, assuming the calcein leaked at the same rate as other components. Calcein fluorescence is highly Stoke’s shifted, and when excited by light at 460 nm, its emission maximum is generally taken to be at approximately 520 nm (Allen and Cleland 1980a). At high concentrations, the fluorescence intensity is small with maxima at ~ 564 and 525 nm, whereas at low concentrations, the maximum shifts to ~ 536 nm. The presence of two peaks in the higher concentration solutions (> 20 mM) is probably due to two environments for the fluorophores corresponding to two different stacked geometries (e.g., J and H aggregates). The wavelength of maximum fluorescence plotted as a function of concentration (Fig. 4c) gives a means of identifying calcein concentration in the solution. Between ~ 10 and 45 mM there is an approximately linear relationship between 1/*λ* and the concentration.

5 $$ {\lambda}_{\mathrm{max}}=-7\times {10}^{-7}\left[\mathrm{calcein}\right]+0.0018 $$

At concentrations, above 50 mM, the fluorescence is very weak and the solutions are very dark. In the context of liposome leakage, the wavelength dependence of calcein fluorescence has the advantage over the usually used intensity measurements of directly enabling a quick estimate of liposome integrity. As the chromatography step of producing calcein filled liposomes in a calcein-free solution produced samples with different total amounts of calcein, referencing the calibration to the total calcein present in an experiment is essential. This can be done by rupturing remaining vesicles at the end of a leakage experiment (usually about 30 min) using detergent (e.g., 0.1% *w*/*v* Triton X-100) to give the maximum possible fluorescence signal.

## Effect of flow on liposome integrity

**Fig. 5:**
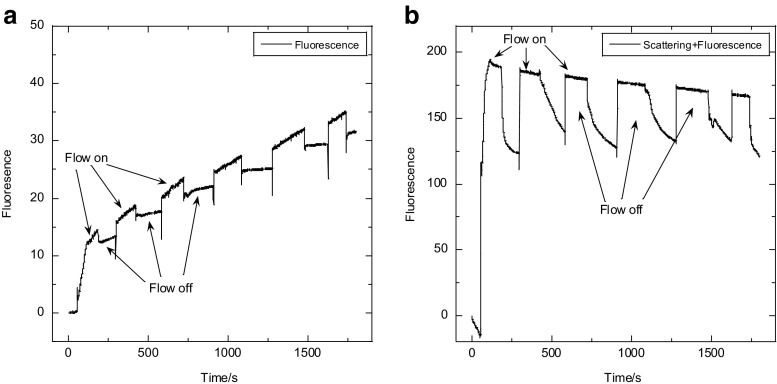
Couette shear flow on (3000 rpm)/off time courses. **a** Fluorescence collected at 180° with a long-pass cut-off filter and **b** fluorescence + scattering measured at 90°, for calcein (50 mM initial concentration inside liposomes) and soybean PC liposomes (20 mg/mL). Data collected as for this figure

**Fig. 6:**
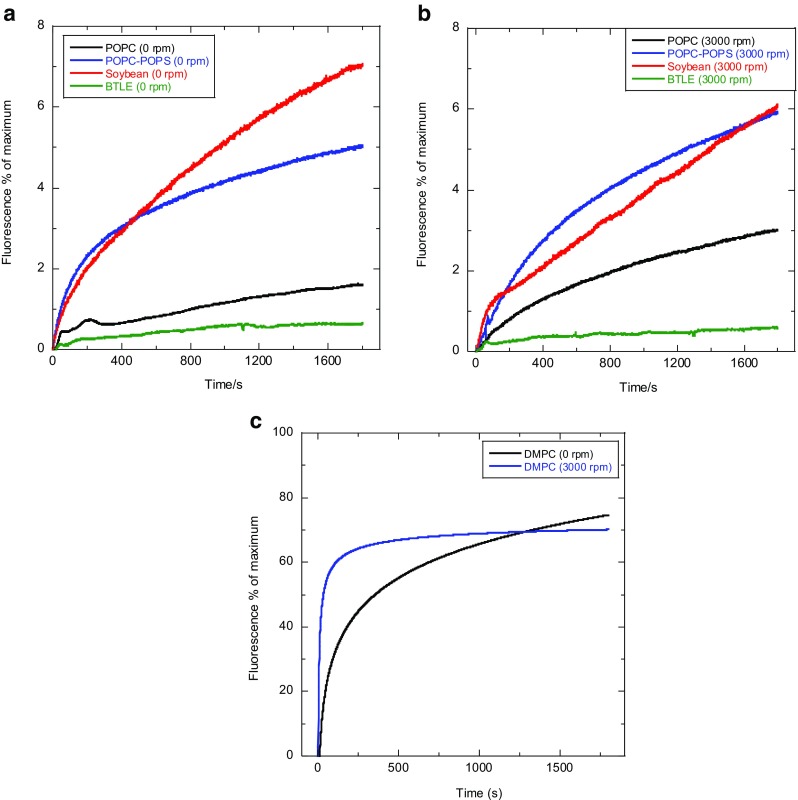
Fluorescence of calcein leaked from a variety of liposomes preparations (see Figs. 2 and 3) held in the microvolume capillary LD cell (Crystal Precision Optics, Rugby, UK) with a 480-nm cut-off filter and 180° detection, with and without the influence of shear flow as indicated in the figures. **a** Stationary samples. **b** Samples in Couette flow. **c** Traces for pure DMPC liposomes fitted to a curve, since measured values had poor signal:noise. Initial calcein concentration inside liposomes was 50 mM, lipid concentration, ~ 20 mg/mL; path length, 0.5 mm. Data collected on a Bio-Logic MOS-450 spectrometer (Bio-Logic, Claix, France)

A series of on/off shear flow time-course experiments measuring both fluorescence and light scattering produced from calcein filled liposomes is shown in Fig. 5. When calcein was only present (at high concentration) inside the liposomes, its fluorescence is approximately zero; however, when it leaks out, it is diluted and has significant fluorescence. Regarding the changes in fluorescence only (Fig. 5a), there is a clear fast (< 1 s) switch between intensity gradients when the shear flow is changed, reflecting calcein changing its average concentration. By way of contrast, when we measure scattering + fluorescence intensity (Fig. 5b), which also directly reflects geometry change, each time the flow ceases there is a slow decrease in signal followed by a fast recovery as flow starts. Flow transformations such as tank-treading, tumbling, or trembling (Lebedev et al. 2008) should cease with the flow, and therefore will not demonstrate this phenomenon. Another feature of the fluorescence-only data of Fig. 6a is that there are two intensity gradients depending on whether flow is on or off. This implies that there is a different leakage mechanism for each state. We therefore can conclude that what we are observing is deformations of the liposomes under flow, as they elongate from a near-spherical shape under stationary conditions to ellipsoidal. The slow timescale reflects molecular rearrangements in the liposome membrane, most likely involving a recovery of liposome contents via slow diffusion of external medium through the lipid membrane whereas the expulsion is much faster by a different mechanism.

## Liposome volume loss in Couette flow

There are various ways to configure fluorescence experiments, but as a complement to microvolume Couette flow LD experiments, we found that 180° detection with a high quality 480-nm cut-off filter (e.g. Hoya Y-50 long-pass filter (Hoya, Santa Clara, CA, USA) in the transmission path after the sample enables only calcein fluorescence to be detected (Fig. 5) (Wemyss et al. 2018), whereas a second photo-detector at 90° (with no filter) detected both fluorescent and scattered incident light analogous.

It is apparent that most of the stationary liposome samples of Fig. 6 leak to some degree and all samples leak more under flow. Pure POPC, soybean PC and BLTE retained most of their integrity whilst stationary and exhibited only a small volume losses under steady shear flow. DMPC (a very common model membrane system used in biophysical experiments) by way of contrast leaked most of its contents within 500 s when stationary, and much more quickly in flow. To ascertain the volume loss explicitly caused by the distortion of the vesicles under flow what needs to be considered is not simply the volume loss under flow (~ 3.6% for POPC), but rather the additional loss (1.4% for POPC) induced in flow compared with non-flow leakage. The volume loss data can be used to estimate sizes of ellipsoids upon making some assumptions about whether surface area changes or not upon initiation of flow.

## Geometry of liposomes in shear flow

**Fig. 7:**
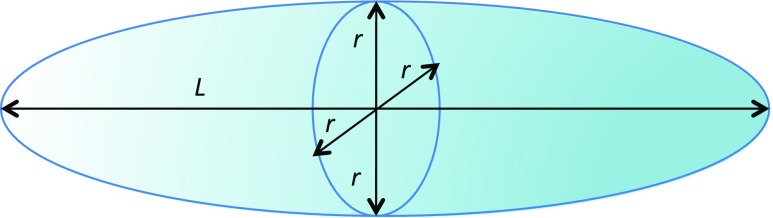
Ovoid model of liposomes used herein: length *L*, radii of both other axes are identical and denoted *r*

Once we know how much volume is lost from a liposome, if we know the shape of the flowing particle, we can deduce bounds on geometric parameters. If we assume that liposomes are spherical at rest and can be represented as an elongated ovoid when in shear flow (Fig. 7), then, if the surface area stays constant when the liposome is distorted in flow (i.e. the lipids are packed to the same density) but the volume changes, we get one limit (Table 1). Table 2 gives the results for the experimental liposomes of Fig. 6 if the surface area is fixed and when we allow the liposomes to expand enough to give the literature value of *S* = 0.030 for bacteriorhodopsin and soy-PC liposomes (Rajendra et al. 2006) using rigid rod calculations of *S.*(McLachlan et al. 2013a) Modelling the liposomes as rigid ovoids/prolate spheroids, gives the not unexpected result that to account for our volume loses and the orientation of the liposomes, flow induces both a change in shape and a change in lipid density due to surface area expansion. Mader et al. (2006) noted similar axis ratios from about 1.2–2.5; however, there microscopy was on large liposomes (10 μm) in low shear rates. The 100-nm vesicles of this work, unfortunately hit the resolution limit of microscopes so no equivalent pictures are available.

**Table 1:** Values of orientation parameter, *S*, and volume, *V*, calculated according to reference (McLachlan et al. 2013b) for ovoids of length, *L*, and radius, *r*, with the same surface area as a sphere of radius 50 nm and volume 524,000 nm^3^. This makes an upper bound for volume change induced by shear flow and a lower bound for *S*

*r*/nm	*L*/nm	Axis ratio	*V*/nm^3^	Fraction *V* loss	*S*
49	104	1.06	5.23 × 10^5^	0.001	0.018
45	121	1.34	5.12 × 10^5^	0.02	0.020
42	134	1.60	4.96 × 10^5^	0.05	0.024
38	154	2.03	4.67 × 10^5^	0.11	0.030
35	171	2.45	4.39 × 10^5^	0.16	0.038
32	191	2.98	4.09 × 10^5^	0.22	0.050
26	240	4.62	3.40 × 10^5^	0.35	0.090
20	316	7.90	2.65 × 10^5^	0.49	0.17
15	423	14.1	2.00 × 10^5^	0.62	0.29
10	636	31.8	1.33 × 10^5^	0.75	0.44
5.	1270	127	6.67 × 10^5^	0.87	0.67

**Table 2:** Volume loss of liposomes in flow determined from the calcein leakage data of Fig. 6. Italic numbers indicate input (experimental or selected for modelling) data and include the assumption of *S* = 0.03 for soy-PC from reference (Rajendra et al. 2006)

Lipid	Fraction volume loss	Surface area/nm^3^	*r*/nm	*L*/nm	Axis ratio	*S*
BTLE	*0.004*	*31,410*	48	108	1.12	0.018
Soy-PC	*0.006*	*31,410*	47	110	1.16	0.018
POPC	*0.014*	*31,410*	46	117	1.27	0.020
POPC/POPS/chol	*0.017*	*31,410*	45	118	1.29	0.020
BTLE	*0.004*	*33,710*	44	138	1.58	0.028
Soy-PC	*0.006*	33,710	43	144	1.70	*0.030*
POPC	*0.014*	*33,710*	39	160	2.04	0.037
POPC/POPS/chol	*0.017*	*33,710*	38	168	2.22	0.040

## Options for correcting for scattering contributions to flow LD spectra of liposomes

Although scattering that occurs in LD experiments is both forward and not-forward, we are saved from having to consider the coherent forward scattering (Mikati et al. 1987; Craig and Thirunamachandran 1984; Long 2002; van de Hulst 1981), as the detector catches this light. To date, only fairly simple corrections to the scattering from the liposomes in flow spectroscopy experiments have been implemented, though some fairly sophisticated theoretical approaches may be found buried in the literature. The simplest correction which seems to improve data for liposomes to correct for scattering in LD spectra is (Nordh et al. 1986)

6 $$ {\tau}_{\mathrm{LD}}=a+b{\lambda}^{-g} $$

Although Eq. (6) has been previously used with apparent success (Beevers et al. 2010; Mikati et al. 1987), a wide variation of values for *g* are required even for similar spectra, in addition to potential for heavily over-correcting at low wavelengths (< 230 nm).

Eq. (6) is reminiscent of Rayleigh scattering which is the elastic scattering of light by particles much smaller than the wavelength of the radiation. With Rayleigh scattering, *g* = 4. However, our liposomes are comparable in size to the wavelength of the light we use to study them and empirically, we find *g* < 4. As outlined below, Gans and Debye modified Rayleigh scattering giving the Rayleigh-Gans-Debye (RGD) approximation (Meeten 1981; Mikati et al. 1987; van de Hulst 1981). It has been used theoretically to model light scattering in a much more sophisticated manner than Rayleigh scattering or Eq. (6). However, as noted by Nordén et al. (Ardhammar et al. 2002) these classical scattering theories assumes that particles are isotropically oriented and solid, but liposomes in flow are anisotropic and hollow. The literature contains what is needed to proceed further for LD experiments, though it has yet to be applied to remove *τ*_*LD*_ to give *A*_LD_ from an experiment with significant scattering occurring.

## Axiss systems

**Fig. 8:**
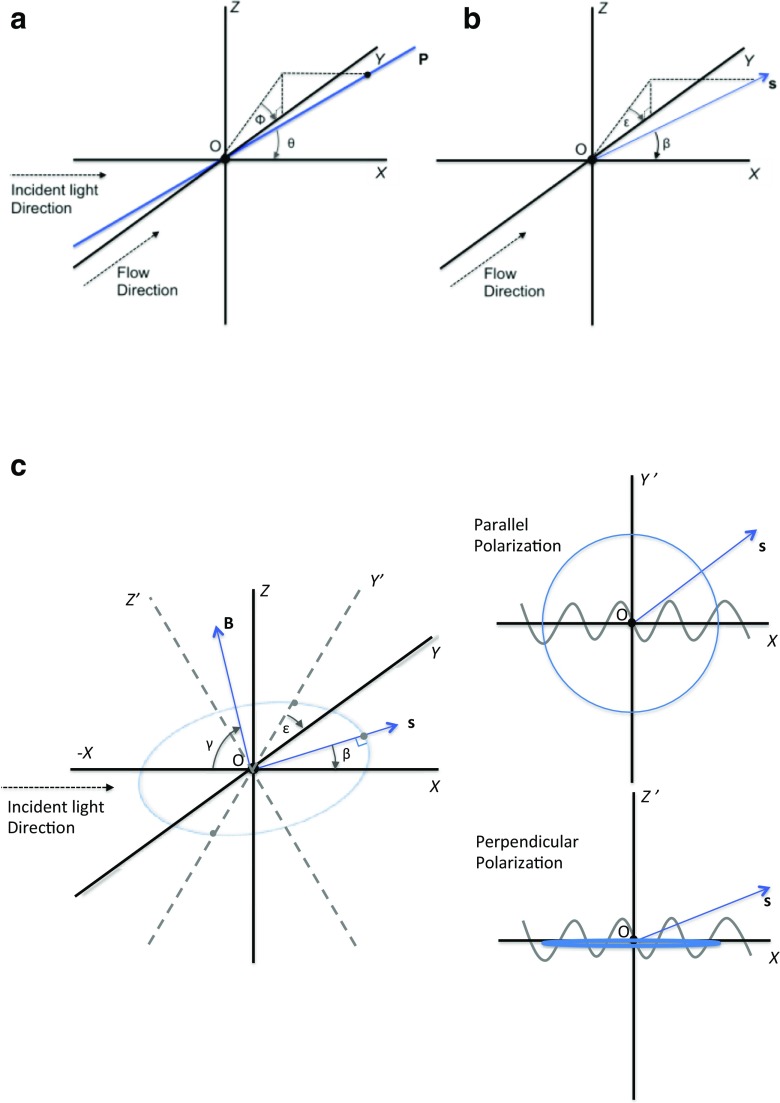
Schematic of an LD experiment for a particle **P** which scatters a photon along vector ***s*** together with geometry definitions used in this work. **a** Particle orientation axis and related polar and azimuthal angles of the particle orientation in space (*ϕ*, *θ*). **b** Scattering vector **s** with associated angles (*β*, *ε*). **c** Schematic diagram of the scattering plane and τ_LD_ axis system {*X*, *Y*^'^, *Z*^'^}; the bisectrix of ***s*** is given by angle γ bounded by XOB

In endeavouring to understand LD, it is necessary to relate the laboratory fixed axis system, {*X*, *Y*, *Z*} to the molecular axis system {*x*, *y*, *z*} where *z* is the orientation axis of the molecule, and *y* is usually taken to be the axis perpendicular to *z* that is best oriented (Nordén et al. 2010). If the orientation is uniaxial, then *x* and *y* are equivalent (Nordén et al. 2010). When writing equations for *τ*_LD_, it is convenient to consider a further axis system, {*X*, *Y*^'^, *Z*^'^}, for the scattering vector for each scattered photon. In this case, *X* is the direction of the incident light (which is the same as the *X* of the laboratory fixed axis system), *Y*^'^ is perpendicular to the plane defined by *X* and the propagation vector of the scattered light (***s***, Fig. 8), and *Z*^'^ makes a right-handed axis system. *ε* is the angle of rotation about *X* that takes {*X*, *Y*^'^, *Z*^'^} into {*X*, *Y*, *Z*}, *β* is the angle between ***s*** and *X*, *ϕ* is the azimuthal angle between the *YZ* projection of *z* and *Y*, and *θ* is the angle between *z* and *X.* The orientation of a particle is defined for the purpose of calculating scattering in terms of the polar angles (*ϕ*, *θ*). (It should be noted that this definition of *θ* is different from the standard LD use, where it is often used to denote the angle between *z* and *Z.*)

## Rayleigh-Gans-Debye theory applied to LD

The scattering approach of Mikati et al.(1987) uses the basic principles for ovoids described by the Rayleigh-Gans-Debye (RGD) theory as outlined by van de Hulst (van de Hulst 1981), with modifications from Meeten (1981) to take into account the polarisation of incident light, and Mikati et al.(1987) went a step further and calculated the ratio of *τ*_LD_ to the isotropic scattering (the scattering depolarisation ratio). Mikati et al.(1987) did not account for the shape and hollow nature of liposomes but work by van de Hulst (van de Hulst 1981) can be included to do so if required. The expression for τ_LD_ derived by Mikati et al.(1987) is

7 $$ {\tau}_{LD}=\frac{1}{4\pi \left(\cos \mu +1\right){r}^2}{\int}_0^{2\pi }{\int}_0^{\pi}\left({I}_Z-{I}_Y\right)\sin \beta \mathrm{d}\beta \mathrm{d}\varepsilon {\int}_0^{2\pi }{\int}_{\mu}^{\pi }f\left(\phi, \theta \right)\sin \theta \mathrm{d}\theta \mathrm{d}\phi $$

where *μ* is the angle of light collected by the detector (cos*μ*~1 for small *μ*), *f*(*ϕ*, *θ*) is a function describing the orientation of the particle, (*I*_*Z*_ − *I*_*Y*_) is the difference in intensities of light scattered from *Z* polarised and *Y* polarised incident light, where *Z* is the orientation (flow) direction and *Y* is perpendicular both to *Z* and the direction of propagation of light. The angles *ϕ* and *θ* are defined in Fig. 8. The Jacobians for our two spherical polar coordinate systems are sin*θ* and sin*β*.

*f*(*ϕ*, *θ*) (and thus *S*) may be calculated to a reasonable approximation for flow LD using the Peterlin-Stuart probability distribution (Peterlin and Stuart 1939) as applied in McLachlan et al. (2013b). The strict definition of *f*(*ϕ*, *θ*)d*s*is the probability that a particle will be found in the solid angle d*s* around the orientation axis *s*. Although strictly *f* is a function of time, the implicit assumption in both Mikati and Meeten et al. (1981) is that time is infinite (in practice large). As the orientation parameter

8 $$ S=\frac{1}{2}\left(3\left\langle {\cos}^2{\theta}_s\right\rangle -1\right)=\frac{1}{2}\left(3\left\langle {\sin}^2\theta {\cos}^2\phi \right\rangle -1\right) $$

with *f*(*ϕ*, *θ*) satisfying the normalisation

9 $$ {\int}_0^{2\pi }{\int}_0^{2\pi }f\left(\theta, \phi \right)\sin \theta d\theta d\phi =4\pi . $$

*S* can be determined from from

10 $$ {\int}_0^{2\pi }{\int}_0^{2\pi }{\sin}^2\theta {\cos}^2\phi f\left(\theta, \phi \right)\sin \theta d\theta d\phi =\left\langle {\sin}^2\theta {\cos}^2\phi \right\rangle . $$

The other part of Eq. (7) which hides complexity are the intensity terms. The intensity of light is related to the electric field by

11 $$ I\left(\omega \right)=\frac{\varepsilon_0{c}_0}{2}n{\left|E\left(\omega \right)\right|}^2 $$

where *n* is the refractive index of the medium, *ω* is frequency, *ε*_0_ and *c*_0_ are the permittivity and speed of light in vacuum. Thus

12 $$ {I}_{\tau }={I}_Z-{I}_Y\propto {\left|{E}_Z\right|}^2-{\left|{E}_Y\right|}^2. $$

Following the literature (Craig and Thirunamachandran 1984; Long 2002; van de Hulst 1981), we express the electric field components of the scattered light in terms of scattering amplitude functions *S*_*i*_ (which depends on the scattering angle, particle geometry, and polarizability) and the electric field of the incident radiation expressed in terms of the {*X*, *Y*^'^, *Z*^'^} axis system.

13 $$ \left(\begin{array}{c}{E}_{Y\hbox{'}}\\ {}{E}_{Z\hbox{'}}\end{array}\right)=\left(\begin{array}{cc}{S}_1& {S}_4\\ {}{S}_3& {S}_2\end{array}\right)\frac{e^{-i\left( kr-\omega t\right)}}{ikr}\left(\begin{array}{c}{E}_{Y_0^{\hbox{'}}}\\ {}{E}_{Z_0^{\hbox{'}}}\end{array}\right) $$

where *k* is the modulus of the wave vector of the light (2π/*λ*, the wavelength in the medium), *ω* is the angular frequency of the light, *r* is the distance of the scattered wave from the scattering centre, ***E*** is the electric field vector of the scattered light, and ***E***_0_ is the electric field vector of the incident light. For small (compared with the wavelength of light) isotropic particles, within the RGD approximation only the amplitudes *S*_1_ and *S*_2_ are non-zero and equate to the polarizations perpendicular and parallel to the scattering plane respectively (illustrated in Fig. 8). (Note that we have chosen to express the ***S*** matrix inverted compared with much of the literature so *Y* and *Z* components of vectors appear in the usual order.)

Although the values o f *I*_*Z*_ and *I*_*Y*_ depend on the dynamic polarizability tensor components, retaining the generality of the anisotropic polarizability leads to complicated equations (Craig and Thirunamachandran 1984) which we end up needing to simplify in order to parametrise the required calculations. We therefore assume the polarizabilities of our particles are isotropic with value *α*. Off-diagonal components of the polarizability tensor are almost always small so this is equivalent to assuming the diagonal components of the polarizability tensor are the same. For liposomes where the molecular components of the scattering particle are a fairly uniform shell, this is reasonable. For other systems, where the orientation is either poor or extremely high, the parametrisation introduced below accounts for anisotropy. Therefore, the ***S*** matrix may be expressed as follows:

14 $$ \left(\begin{array}{cc}{S}_1& {S}_4\\ {}{S}_3& {S}_2\end{array}\right)=i{k}^3\alpha R\left(\beta, \varepsilon \right)\left(\begin{array}{cc}1& 0\\ {}0& \cos \beta \end{array}\right) $$

In the literature, Eq. (14) is commonly found without any explanation of the terms. If the goal is to calculate the scattering for oriented rods or ellipsoids, a level of understanding is required (Mikati et al. 1987; Craig and Thirunamachandran 1984; Long 2002; van de Hulst 1981). *k* accounts for the *S*_*i*_ amplitude wavelength dependence; *α* is the isotropic polarizability of the particle; *R* is described variously as the interference function, form function, or form vector, which accounts for particle orientation and geometry. Since RGD scattering treats every volume element as an independent scatterer, all the waves scattered in a particular direction (*β*, *ε*) interfere because of their different origins in space. *R*(*β*, *ε*) therefore represents a phase factor to correct for these interference effects, relating the phases of all the scattered waves to a common origin.

For the LD experiment, we need to determine the scattered light vector in the *Y*^'^*Z*^'^ coordinate system for incident light polarised along either *Z* or *Y.* The vectors are (where the subscripts denotes the axis system and the argument denotes the original polarisation)

15 $$ {\left(\begin{array}{c}0\\ {}{E}_{Y_0^{\hbox{'}}}(Z)\\ {}{E}_{Z_0^{\hbox{'}}}(Z)\end{array}\right)}_{XY\hbox{'}Z\hbox{'}}=\left(\begin{array}{ccc}1& 0& 0\\ {}0& \cos \varepsilon & \sin \varepsilon \\ {}0& -\sin \varepsilon & \cos \varepsilon \end{array}\right){\left(\begin{array}{c}0\\ {}0\\ {}1\end{array}\right)}_{XY Z}{E}_0 $$

and

16 $$ {\left(\begin{array}{c}0\\ {}{E}_{Y_0^{\hbox{'}}}(Y)\\ {}{E}_{Z_0^{\hbox{'}}}(Y)\end{array}\right)}_{XY\hbox{'}Z\hbox{'}}=\left(\begin{array}{ccc}1& 0& 0\\ {}0& \cos \varepsilon & \sin \varepsilon \\ {}0& -\sin \varepsilon & \cos \varepsilon \end{array}\right){\left(\begin{array}{c}0\\ {}1\\ {}0\end{array}\right)}_{XY Z}{E}_0 $$

The transformation matrix takes the {*Y*, *Z*}coordinate system to {*Y*^'^, *Z*^'^}*.* From Eqs. (12)–(16) for *N* particles it follows that

17 $$ {I}_Z=\frac{N{k}^4{V}^2{\alpha}^2{I}_0}{r^2}{R}^2\left(\beta, \varepsilon \right)\left[{\cos}^2\varepsilon +{\cos}^2\beta {\sin}^2\varepsilon \right]{\alpha}^2={V}_f\frac{{m_1}^2-1}{{m_1}^2+2}+\left(1-{V}_f\right)\frac{{m_2}^2-1}{{m_2}^2+2} $$

18 $$ {I}_Y=\frac{N{k}^4{V}^2{\alpha}^2{I}_0}{r^2}{R}^2\left(\beta, \varepsilon \right)\left[{\sin}^2\varepsilon +{\cos}^2\beta {\cos}^2\varepsilon \right]. $$

### Polarisability, *α*

Mikati et al.(1987) avoided calculating *α* by only determining the ratio of *τ*_*LD*_ and *τ*_*A*_. When *α* is required in RGD calculations, the simple uniform value ((*m* − 1)/(2*π*))*dV* is often used for isotropic particles with a relative refractive index, *m*, close to 1. Liposomes usually do have a similar refractive index to that of the surrounding medium; however, in any work involving hollow particles (as in liposomes), where the refractive index of the particle centre is not identical to that of its outer shell, the polarisability is not constant and *α* needs to reflect this. Application of theory from Yoshikawa et al. (1983) regarding scattering from spherical shells can be applied here to refine *α*:

19 $$ \alpha ={\mathrm{V}}_f\frac{{m_1}^2-1}{{m_1}^2+2}+\left(1-{\mathrm{V}}_f\right)\frac{{m_2}^2-1}{{m_2}^2+2} $$

where *V*_*f*_ is the volume fraction of the lipid in the particle, *m*_1_ and *m*_2_ are the complex relative refractive indices of respectively the shell and inside of the particle. We can estimate that the polarisability of our water-filled liposomes is about 5% higher than that of a solid lipid sphere.

### R form function for solid ovoids and rod-like particles

The form vectors, *R*(*β*, *ε*), depend on the particle shape, size, axis ratio and orientation. The RGD form factor with a wavelength relationship of *k*^4^ is attractively simple. However, it was found not to work for tubulin microtubules (Nordh et al. 1986) (Marrington et al. 2006) and actin microfilaments (Rodger et al. 2006) experimental data and our attemtps to make it work for liposomes also failed. Dupuy and Montagu compared RGD and Mie theory (which has a *k*^2^ dependence, *k* = 2π/*λ*) for liposomes of size 170–300 nm, and found Mie theory together with a constant term described the behaviour better. The expressions used in (Meeten 1981; van de Hulst 1981) for two cases: solid ovoids, with long and short semi-axis lengths of *a* and *b*, respectively, and rod-like particles of length *l*, and diameter *d* have approximately the same *k* dependence.

For solid ovoids:

20 $$ R\left(\beta, \varepsilon \right)=\left(\frac{3\sin {K}_o}{K_o^3}-\frac{3\cos {K}_o}{K_o^2}\right) $$

where

21 $$ {K}_O=2k\sin \frac{\beta }{2}{\left[{b}^2+\left({a}^2-{b}^2\right){\cos}^2\gamma \right]}^{1/2} $$

and *γ* is the angle between *z* (the particle axis) and the vector that lies on the bisector of – *X* and the scattering vector, ***s*** (the so-called bisectrix, expressed in the *XYZ* coordinate system)

22 $$ B={\left(-\sin \frac{\beta }{2},-\cos \varepsilon \cos \frac{\beta }{2},\sin \varepsilon \cos \frac{\beta }{2}\right)}_{XYZ} $$

so

23 $$ \cos \gamma =-\cos \theta \sin \frac{\beta }{2}+\sin \theta \cos \frac{\beta }{2}\cos \left(\phi -\varepsilon \right). $$

Alternatively, for rod-like particles:

24 $$ R\left(\beta, \varepsilon \right)=F\left( kd\sin \frac{\beta }{2}\sin \gamma \right)E\left( kl\sin \frac{\beta }{2}\cos \gamma \right) $$

where

25 $$ F(u)=2\left(\frac{\sin u}{u^3}-\frac{\cos u}{u^2}\right) $$

and

26 $$ E(v)=\frac{\sin v}{v} $$

*K*_*O*_ and the corresponding arguments in Eq. (24) denote the phase shift of the particle (ovoid and rod, respectively) in relation to the particle position and polar/azimuthal angles (*β*, *ε*).

### Incident intensity–distance relationship and accounting for hollow spheres

A key parameter for calculating the scattering is the instrument parameter *I*_0_/*r*^2^. Previous workers have failed to explain exactly how this parameter can be determined and by taking ratios of e.g. LD and absorbance avoided the issue. Determination of *I*_0_ for a particular emitter although complex is possible, however, hard to do accurately in part because of the difficulty in a classical LD experimental setup of determining a value for *r*. An alternative is to parametrise *I*_0_/*r*^2^ via the fitting of a scattering curve to a spectrum with known liposome deformation and orientation.

Parametrising *I*_0_/*r*^2^also accounts for the reduction in value of the form factors due to the liposomes not being made of solid lipid if we parametrise with approximately the same size particles since the general form factor equation is:

27 $$ R\left(\beta, \varepsilon \right)=\frac{1}{V}{\int}_{-\infty}^{\infty }B{e}^{iK} dp $$

where the integration represents ‘slices’ of the particle perpendicular to the bisectrix, of area *B* and thickness *p* (equating to *2b* for ovoids, and *d/l* for rods). Thus, to account for hollow particles, the factor *B* is scaled by the reduction in lipid volume.

### Considerations for semi-rigid particles

Although in this work, we calculate *S* directly following McLachlan et al. (2013b), for completeness and for use in further work where the rigid rod approximation may be inapplicable, the equations for *S* in the three axis systems used in this work are given below. Mikati et al.(1987) also make use of such equations. In spectroscopic applications, *S* is usually calculated in terms of the angles with respect to the flow direction. So, in addition to the angles defined in Figures A1 and A2, we define *Θ* to be the angle between *z* and *Z* and *Φ* to be the angle between the projection of *z* onto the *XY* plane and *Y*. We also follow Mikati et al.(1987) and define hydrodynamic angles where *θ*_*p*_ is the angle between *Y* and *y* and *ϕ*_*p*_ is the angle between the *XZ* projection of *z* and *X*.

We may then write in the {*X*, *Y*, *Z*} coordinate system

28 $$ {\displaystyle \begin{array}{c}\mathbf{p}={\left(\cos \theta, \sin \theta \sin \phi, \sin \theta \cos \phi \right)}_{XYZ}\\ {}={\left(\sin \varTheta \cos \varPhi, \sin \varTheta \sin \varPhi, \cos \varTheta \right)}_{XYZ}\\ {}={\left(\sin {\theta}_p\cos {\phi}_p,\cos {\theta}_p,\sin {\theta}_p\sin {\phi}_p\right)}_{XYZ}\end{array}} $$

From which it follows that the orientation parameter, *S*, may be written in each set of angles as follows

29 $$ {\displaystyle \begin{array}{c}S=\left\langle {\cos}^2\varTheta \right\rangle -\left\langle {\sin}^2\varTheta {\sin}^2\varPhi \right\rangle \\ {}=-\left\langle {\sin}^2\theta \cos 2\phi \right\rangle \\ {}=\frac{1}{2}\left(3\left\langle {\sin}^2{\theta}_p\right\rangle -\left\langle {\sin}^2{\theta}_p\cos 2{\phi}_p\right\rangle -2\right)\end{array}} $$

where 〈〉 denotes average over the particles in the solution. For a uniaxial system, one averages over *Φ*and line 1 of Eq. (29) reduces to the more familiar

30 $$ S=\frac{1}{2}\left(3{\cos}^2\varTheta -1\right) $$

being the orientation parameter for a uniaxial system.

## Application of Eq. (7) to remove scattering contributions to measured LD spectra

As Fig. 6 indicates, POPC forms fairly stable 100 nm liposomes. The LD spectrum for a known volume loss relative to the sphere (established via calcein leakage studies) and a known orientation parameter, lets us determine *I*_0_/*r*^2^ to be between 5 × 10^−12^ (assuming no change in surface area) and 1.05 × 10^−12^ for the experimental 7% surface area increase for soybean PC (assuming *S* = 0.03 as in reference (Rajendra et al. 2006)). Our code for implementing Eq. (7) is given in the supplementary information. The value of *I*_0_/*r*^2^ can be manually altered in our code to evaluate Eq. (7) until e.g. the long-wavelength region (~ 400–500 nm) calculated scattering overlays the experimental scattering and similarly for other regions that do not have any absorbance signals. Once a suitable *I*_0_/*r*^2^ value is set, other samples can be fit in the same manner, using the long axis of the liposome as the variable (Table 3). When the fits are completed the liposome parameters including *S* axis ratio and % volume loss can be determined just from the LD data.

**Table 3:** Vesicle scattering correction input parameters (http://www.liposomes.org/search/label/Number%20of%20lipid%20molecules%20per%20liposome; Kucerka et al. 2011; Kučerka et al. 2011) for the code written to implement Eq. (7) in MatLab.(MATLAB and statistics toolbox release 2014b, 2014)

Input parameter	Value
Lipids per vesicle	8.17 × 10^4^
Lipid membrane thickness/nm	4
Capillary volume/μl	83.25 × 10^−6^
Capillary outer diameter/mm	2.9
Capillary inner diameter/mm	2.4
Shear rate/s^−1^	1.8 × 10^3^
Medium refractive index	1.33
Vesicle refractive index	1.42
Incident light wavelength range/nm	As in experiment
Distance from detector/cm	3
Detector radius/cm	1.5
*I*_0_/*r*^2^	1.05 × 10^−12^

**Fig. 9:**
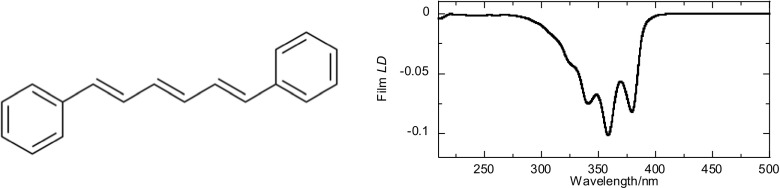
Molecular structure of the fluorescent chromophore DPH and its stretched film LD spectrum (DPH was dropped from a concentrated solution in CHCl_3_ onto prestretched polyethylene film) (Razmkhah et al. 2014)

In order to have an *A*_LD_ signal for some test samples Fig. 3, data are for liposomes with diphenyl hexatriene (DPH) (Dupuy and Montagu 1997) incoporated in the membranes as a spectral reporter. DPH is a long thin hydrophobic molecule whose film LD spectrum in polyethylene (Fig. 9, *S*~0.5) demonstrates that the 360 nm region is polarised along the long axis of the molecule. Thus, since our lipids do not absorb at 360 nm, DPH acts as a marker to detect the orientation of lipids via LD, with the negative signal seen at 360 nm indicating DPH is indeed inserted parallel to the membrane normal (Eq. (3)). The magnitude of the DPH LD relative to the absorbance of the same sample indicates the degree of orientation of the liposome, though it is clear that different lipids incorporate different amounts of DPH. The fixed surface area scattering corrections illustrated in Fig. 3 give a reasonable estimate of *A*_LD_, but it is clear that flow stretches the liposomes and induces more scattering than this lower bound indicates. Despite the assumptions, we have made to determine parameters for our RGD implementation for LD our pragmatic approach to determining parameters to implement established scattering theory works well for different liposomes with low leakage rates when modelled as ellipsoids as summarised in Table 2.

Rod-shaped (rather than ovoid) fits were also attempted for the same liposome data, however, although similar fits could be obtained at higher vesicle concentrations, rod-fitting did not work well at low concentrations and was therefore rejected in favour of purely ovoid scattering.

## Conclusions

In this work, we have reviewed literature scattering theory for flow LD spectroscopy and illustrated an implementation of the Rayleigh-Gans-Debye scattering theory. We have relied on the expansion of RGD theory undertaken by van de Hulst (1981), Meeten (1981), and Mikati et al.(1987). We used the rigid rod Peterlin-Stuart probability distribution calculation of the orientation parameter, *S*, to calculate the turbidity linear dichroism of liposomes. The combination enables the absorption linear dichroism even in the presence of significant scattering to be determined. Values for the degree of deformation and volume loss in the LD experiment is a by-product of the spectral correction process. Thus we only need to do the calcein fluorescence assay for the calibrating liposome sample. In this work, we selected a stable set of liposome deformation data to act as a standard from which to calculate instrument parameters *τ*_*LD*_ for any lipid system collected in that instrument configuration. Thus, for the first time, we showed that literature theory can be applied to extract the true absorbance LD spectra of light scattering samples, rather than only ratios.

Although the focus in this review has been almost exclusively on flow LD spectroscopy, any optical spectroscopy technique which is less complicated can be addressed with the same approach. The differential intensity term would in general be replaced by an isotropic incident intensity term and we would not need to worry about flow-induced particle volume changes during the experiment.

According to Rayleigh theory, the relationship that a particle has with the wavelength of the incident light has a *k*^4^ dependence. However, it has been previously shown that this power relationship does not fit experiment for liposomes. Instead the relationship that is demonstrated is far closer to that of classical Mie theory, with a *k*^2^ dependence. Since our particles are approaching the limits of the size restriction outlined by RGD theory, and just barely under the minimum for Mie theory, it is not unreasonable to see elements of both in effect. The main differences in the approximations for each approach are the particle shape factors, since Mie is primarily restricted to spherical particles whereas RGD allows for asymmetrical particles, and a constant term. The parametrisation approach taken here allows us to take into account unknown constants (e.g. stray light, inhomogeneity, hollow particles)) into the *I*_*0*_/*r*^2^ term. Therefore, what we have illustrated is a quasi RGD-Mie theory instead of strictly classical RGD.

A calcein fluorescence intensity assay to measure volume loss from liposomes when stationary and when deformed under shear flow was also illustrated. Calcein fluorescence not only enhances significantly with dilution as is well established but also shifts from 525/563 nm to 536 nm. 1/*λ*_max_ can be used to determine calcein concentration below ~ 45 mM with fluorescence depending approximately linearly on concentration between 45 and 10 mM. Leakage depends upon the type of lipid and the duration of flow, with the shorter chain DMPC exhibiting the highest degree of leakage even when stationary, questioning the usefulness of the extensive DMPC liposome work in the literature for kinetics and comparison studies.
